# Investigation of association between maternal 25-OH vitamin D serum levels and neonatal early onset sepsis in newborns by evaluating key factors

**DOI:** 10.1186/s12944-019-1095-3

**Published:** 2019-07-13

**Authors:** Maryam Saboute, Rahman Yavar, Mandana Kashaki, Fatemeh Kazemi Khaledi, Nasrin Khalesi, Farzaneh Rohani

**Affiliations:** 1 Shahid Akbarabadi Clinical Research Development Unit (ShACRDU), Iran University of Medical Sciences (IUMS), Tehran, Iran; 2 Department of genetics, Akbarabadi Hospital, Iran University of Medical Sciences, Tehran, Iran; 3 Department of Pediatrics, Ali Asghar Hospital, Iran University of Medical Sciences, Tehran, Iran; 4 Maternal, Fetal and Neonatal Research Center, Tehran University of Medical Sciences, Tehran, Iran; 5 Pediatric Growth and Development Research Center, Iran University of Medical Sciences, Tehran, Iran; 6 Department of Pediatric Endocrinology and Metabolic Diseases, Mofid Children Hospital, Shahid Beheshti University of Medical Sciences, Tehran, Iran

**Keywords:** Maternal, Vitamin D, Neonatal early onset sepsis, Vitamin D supplement, Apgar score

## Abstract

**Background:**

The goal of this study was to evaluate the relationship between maternal 25-OH Vitamin D serum levels and neonatal early-onset sepsis in newborns by the effective factors.

**Methods:**

A case-control study was done and 64 neonates hospitalized in Akbar Abadi Hospital (Tehran- Iran; 2016) and their mothers were enrolled. The case group consisted of 32 NICU term hospitalized neonates due to neonatal early-onset sepsis. Thirty-two term newborns that referred to hospital for rule out hyperbilirubinemia during the first 72 h of life were also considered as the control.

**Results:**

Sixty- four mothers with mean age 28.76 ± 6.60 years and mean gestational age 39.64 ± 1.62 weeks entered the study. There was a significant correlation between sepsis and older age of mothers and low Apgar score (*P*-value = 0.02, 0.01 respectively). The maternal vitamin D serum level was reversely correlated with neonatal sepsis occurrence (*P*-value = 0.03). There was a significant correlation between maternal vitamin D supplement intake during pregnancy and lower risk for neonatal sepsis (*P*-value = 0.003).

**Conclusion:**

The level of maternal serum Vitamin D was inversely correlated with neonatal sepsis occurrence and intake of vitamin D supplement during pregnancy could decrease the risk of early neonatal sepsis.

## Background

Neonatal early-onset sepsis (NEOS) defines as systemic bacteremia with positive blood or cerebrospinal fluid culture in the first 7 days of neonate’s life. It is a serious complication with relatively low incidence (0.5–1.2 cases per 1,000 live births) and high morbidity/mortality rates [1–3]. Several maternal and neonatal causes including the colonization of organisms in the genitourinary tract, invasive procedures during pregnancy, prolonged rupture of membranes, instrumental delivery, prematurity, low birth weight, neonatal anomalies, and low Apgar scores are involved in the etiology of NEOS. Group B Streptococcus, *Escherichia coli*, and *Listeria monocytogenes* are also reported as the most frequent pathogens in NEOS [4, 5]. Different studies demonstrated that low 25-hydroxyvitamin D3 (25 (OH) D) or calcidiol, levels are linked to low birth weight and gestational age. Moreover, low maternal 25 (OH) D levels effect on the Apgar score of the newborn [6].

Recent studies have indicated a correlation between maternal serum vitamin D status and health consequences for neonates, it has been indicated that maternal 25OHD is markedly associated with offspring birth weight, where vitamin D controlling gestational age plays a key role in mediating the effect of 25 (OH) D on birth weight. There has been a growing body of evidence that indicated gestational 25 (OH) D insufficiency can be associated with adverse pregnancy outcomes [6]. Increasing evidence from a meta-analysis of randomized controlled trials has helped to elucidate the role of D supplementation on maternal and neonatal results, where at pregnancy time the vitamin D supplementation appear to be related to a higher circulating 25(OH) D levels, birth length and birth weight. Vitamin D deficiency may be involved in the pathogenesis of sepsis and sepsis-related disseminated intravascular coagulation (DIC) [7]. Vitamin D has also been found to have some neonatal protective properties against infectious diseases by regulating of the innate-adaptive immune system, anti-inflammatory effects, increased monocyte responses, and enhanced mucosal barrier as well as by endothelial function [8–10]. There is close and deep relationship between maternal and fetal vitamin D [11].

Vitamin D deficiency is a worldwide complication with prevalence ranging from 18 to 84% that is more dependent upon geographic region, ethnicity, type of clothing and dietary intake. The highest prevalence rate (60–80%) is reported among high-risk population such as pregnant women, subjects with low dietary vitamin D intake, pre-pregnancy obese women and individuals with limited sun exposure [12, 13]. Various studies have shown that the difference in free vitamin D has an effect on DBP levels, but the effect of vitamin D supplementation has not been established [11].

Although there are a few studies that assessed correlations among maternal serum vitamin D status and neonatal sepsis. Another approach to try to clarify the effect of vitamin D supplementation is to design better-designed randomized controlled trials assessing clinically relevant outcomes. it seems that more approaches are needed in this field. There is a growing of evidence that sufficient vitamin D-supply of mother and child can play a pivotal role in preventing birth complications [11]. It is noteworthy that free 25(OH) D measuring has been recently demonstrated to be favorable laboratory parameter for monitoring vitamin D during pregnancy [14]. Therefore, measurement of free 25 (OH) D should be considered for different concentrations of DBP, including pregnancy. It should be taken into consideration that the 25 (OH) D measurements are relatively cost-effective, timely and robust [11, 14].

Our results can provide some informative data related such correlations among Iranian mothers with a specific type of clothing, ethnicity, geographic region and high intake of vitamin D [12, 13, 15, 16]. Therefore, the present study was planned to investigate the relationship between maternal 25-OH Vitamin D serum levels and neonatal early-onset sepsis in newborns by the effective factors.

## Materials and methods

### Study design

A case-control study was carried out in the NICU of Akbar-Abadi Hospital affiliated to Iran University of Medical Sciences (Tehran-Iran) from 2016 to 2017. Sixty-four term neonates and their mothers have entered the study.

### Patients

Neonatal early-onset sepsis (case group; NEOS) consisted of 32 terms NICU hospitalized neonates as suspected sepsis in the patients’ group. EOS diagnosis was considered based on clinical and laboratory findings during the first 72 h of life to have a high potential NEOS according to the criteria defined by Soliman Gamal et al., 2017, such as temperature instability, heart rate dysrhythmia, poor feeding or intolerance, weight loss, abdominal distension, and necrotizing enterocolitis, and respiratory clinical signs containing distress, supplemental oxygen, mechanical ventilation requirement, apnea, tachycardia or bradycardia, and hypoxemia, as well as positive blood culture, elevates C-reactive protein (CRP) and hematologic cell blood count findings [17].

### Controls

In the control group, thirty-two healthy neonates with no prenatal risk factor for NEOS were enrolled. Whole neonates were subjected to complete history taking containing gestational age, maternal age, sex, type of delivery, maternal vitamin D concentration, birth weight, Apgar scores, the birth season and neonatal activity (doing or not doing well). Term newborns that referred to the hospital for ruling out hyperbilirubinemia during the first 72 h of life were also considered as the control group. Both groups were adjusted regarding their ages. Moreover, all neonates had breastfeeding during the time of the study.

Inclusion criteria were normal birth weight (> 2500 g), term birth and suspected with sepsis. It should be taken in to account that sepsis is a bacterial infection were confirmed by a positive culture before the baby is 1 month old.

Exclusion criteria were included; Maternal poor bad obstetric history correlated to neonatal sepsis including PROM, Chorioamnionitis, congenital anomalies, insulin dependent diabetes mellitus (IDDM), maternal systemic and chronic disease, hematologic disorders, use of medication, twin pregnancy, drug abuse, as well as mother’s death before assessing of vitamin D serum level.

Demographic characteristics and clinical data of neonates and mothers including maternal age, level of education, geographic region, perinatal complications, history of using supplement during pregnancy, neonatal birth weight, gestational age, sex, first minute Apgar score, the season of birth were recorded in some checklists.

### Laboratory evaluation

For determining maternal vitamin D status of both groups, 5 ml of mother’s blood was collected, labeled and afterward sent to the laboratory to measure 25-OH vitamin D serum level by ELISA Kit (bioMérieux, France). In addition, vitamin D insufficiency was defined as a serum concentration of vitamin D < 30 ng/ml [18].

Based on an investigation by certinkaya et al. [19], the mean vitamin D serum concentrations among EOS suspected and unsuspected newborns were determined as 21.7 ± 6.5 and 27.1 ng/ml (CI = 8.7), respectively. Using the following formula and proposed sample size of 32 for each group, our study had a power of 80% and an alpha error of 0.05.

$$ \mathrm{n}=\frac{{\left({\mathrm{Z}}_{\left(1\hbox{-} \upalpha /2\right)}+{\mathrm{Z}}_{\left(1\hbox{-} \upbeta \right)}\right)}^2\left({{\mathrm{sd}}_1}^2+{{\mathrm{sd}}_2}^2\right)}{{\mathrm{d}}^2} $$

### Statistical analysis

The statistical analyses were performed using software package SPSS version 18. Quantitative and qualitative variables were reported by mean ± SD and percent, respectively. Student t-test and Mann-Whitney test were utilized to compare among groups for comparing quantitative data. Furthermore, Chi-square and Fischer exact test were used to analyzing the correlations between qualitative variables. Additionally, One-way ANOVA and logistic regression analyses were performed to analyze the correlations between variables. The level of significance was considered as *P* < 0.05.

## Results

Sixty-four mothers with a mean age of 28.76 ± 6.60 years and mean gestational age of 39.64 ± 1.62 weeks have entered the study. Forty-one mothers had a normal vaginal delivery and 23 had a cesarean section. Among all enrolled mothers, 16 subjects exhibited perinatal complications including hypothyroidism and diabetes, while 23 did not receive vitamin D supplement during pregnancy (20 mothers consumed vitamin D supplement for more than 3 months). The mean maternal serum vitamin D was determined as 25.72 ± 12.37 (Min: 2, Max: 51.8), (Table 1).

**Table 1:** Basic demographic characteristics of the mothers and their newborns

Characteristic (*n* = 64)	
Maternal age	28.76 ± 6.60
Educational Level	Diploma and upper: 37.5
	Lower diploma: 62.5
Maternal Vitamin D concentration (nmol/L)	25.72 ± 12.37
Gestational age	39.64 ± 1.62
Birthweight	3002.96 ± 521.74
Type of delivery	
NVD^a^	64.06
C/S^b^	35.93

^a^vaginal delivery

^b^cesarean section

Of all 64 neonates, 30 subjects were male and 34 were female. The mean birth weight was 3002.96 ± 521.74 g. The lowest and highest Apgar score at first minute were determined to be 6 and 9, respectively.

Our results revealed that the mean maternal age in the case group was significantly higher than the control group; older mothers had a greater chance of having a neonate with EOS (*p* = 0.004). Neonatal sepsis was more common in males when compared with females (*P* = 0.003). A significant correlation was also observed between neonatal sepsis and low first minute Apgar score (*P* = 0.003). The level of maternal serum vitamin D was reversely correlated with sepsis occurrence (*P* = 0.03). Furthermore, there was a significant correlation between maternal vitamin D supplement intake during pregnancy and lower risk for neonatal sepsis (*P* value = 0.003). An increasing body of evidence suggested that maternal complications like hypothyroidism and gestational diabetes could increase the risk of neonatal sepsis (*P* = 0.02). Although vitamin D insufficiency was less common among mothers who spent their pregnancy during spring and summer as compared to those with a fall and winter pregnancy, the difference of vitamin D status between groups was not found to be significant (*P* = 0.09). Type of delivery gestational age and neonatal birth weight were not also significant determining factors for neonatal sepsis (*P* > 0.05).

**Fig. 1:**
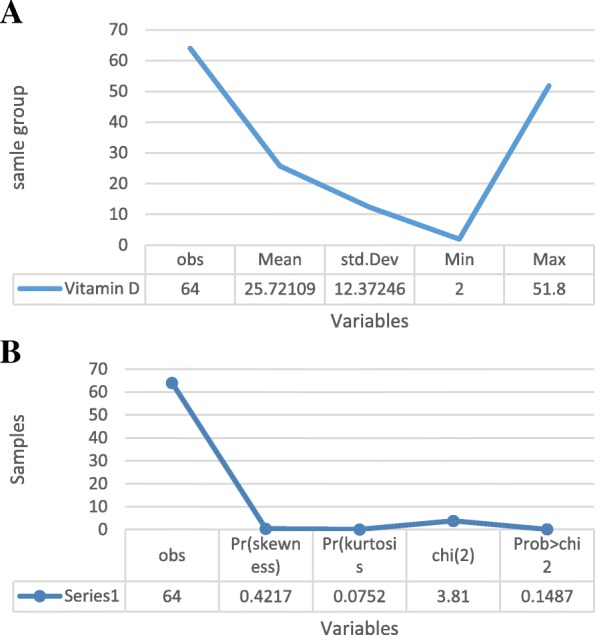
The detail of vitamin D level in both sepsis and non- sepsis groups: **a** Vitamin D levels the sepsis and non-sepsis group **b** Normal distribution of vitamin D levels the sepsis and non-sepsis group

We used the binary logistic regression to determine interactions between variables. Sepsis was considered as dependent variable, while maternal age, serum Vitamin D level, gestational age, first minute Apgar score and perinatal complications were considered as independent factors (Table 2) Among all evaluated factors, older mothers and low Apgar score affect the incidence of neonatal sepsis (*p* = 0.02; *p* = 0.01). The detail of vitamin D and other variables displayed in the Fig. 1 Prediction value for related risk factors used to estimate the coefficient of determination based on the linear regression model. Based on Cox and Snell model, 63.7% of neonatal sepsis could be predicted by aforementioned variables The regression coefficient was confirmed based on the Cox and Snell mod as 0.637, where indicates that the change of the response variable (sepsis) is presented by predictor variables.

**Table 2:** Descriptive data of mother-infant according to suspected sepsis and non-septic subjects

Data of mothers and newborn (*n* = 64)	Suspected sepsis	Non-septic sepsis	*p*-value
Maternal age	Skewed: − 0.851 ± 2	Skewed: 0.802 ± 2	0.004;
	Kurtosis: −0.214 ± 2	Kurtosis: 0.154 ± 2	OR = 2.12
First minute Apgar score (mean ± SD)	8.5 ± 8.80	8.93 ± 0.24	0.003; OR = 1.19
Type of delivery (%):			
NVD	50	78.13	0.32
C/S	50	21.88	
Birth weight	Skewed: 0.731 ± 2	Skewed: 0.861 ± 2	0.249
	Kurtosis: 0.094 ± 2	Kurtosis: 0.090 ± 2	
Maternal Vitamin D	Skewed: −0.901 ± 2	Skewed: −0.930 ± 2	0.034; OR = 2.92
concentration (nmol/L)	Kurtosis: −0.103 ± 2	Kurtosis: − 0.089 ± 2	
Sex (%)	Male: 46.87	Female: 53.13	0.003; OR = 3.27
Gestational age	3.39	1.71	0.078

## Discussion

In the present study, we evaluated the correlation between maternal serum vitamin D and neonatal early-onset sepsis. Based on the results presented herein, the level of maternal serum vitamin D was inversely correlated with neonatal sepsis occurrence. It seems that vitamin D has protective effects against neonatal sepsis.

Vitamin D has been found to play a key role in increasing defense against bacterial and viral agents by triggering the production of the antimicrobial peptide and has been found to trigger its immunomodulatory role by suppressing inflammatory cytokines such as interleukin-6 (IL-6), [20]. 1a,25(OH)2D is capable of increasing the antimicrobial properties of monocytes and macrophages as well as play a role in increasing chemotaxis and the phagocytic properties of macrophages. Moreover, can be involved in the mitigation of adaptive immunity, which is likely to yield benefits in autoimmune afflictions.

A systematic review and meta-analysis by Haan et al. suggested an association between vitamin D deficiency with infection rate, sepsis, and neonatal mortality rate [12]. Although we did not consider the neonatal vitamin D status, other studies suggested that there is a positive correlation among mother and umbilical cord blood vitamin D concentration [21, 22]. Moreover, our previous study has shown a strong positive correlation between maternal serum vitamin D and neonate’s birth weight [23]. It is also supposed that low birth weight newborns are at an increased risk of developing neonatal morbidities like NEOS. In accordance to our results, Yang et al. demonstrated that mothers and their neonates with NEOS had lower 25-OHD levels in comparison with their counterparts in the control group, leading to the neonatal early-onset sepsis risk for full-term infants [24].

Gamal et al. also confirmed a significant negative association between neonatal- maternal 25-OH vitamin D serum levels and sepsis markers in 50 neonates with NEOS [25].

Our findings revealed that at pregnancy time the vitamin D supplementation could decrease the hazard of early neonatal sepsis. In accordance to our results, an investigation by Quraishi et al. indicated that a single bolus dose of 400,000 IU vitamin D in patients with sepsis could quickly increase serum 25OHD concentration, where was capable of modulating the expression level of pro-inflammatory cytokines such as IL-1β and IL-6, resulting in improved clinical outcomes [26]. On the other hand, maternal vitamin D level has been found to be potentially linked to a decreased risk of neonatal infectious diseases including respiratory infections and sepsis [27].

Regarding the maternal demographic factors, we found that the mean maternal age in the case group was significantly higher than the control group. It is supposed that risks of neonatal morbidity are more common among infants of older mothers, but this finding was not confirmed by other investigations; it has been indicated that maternal age < 20 can be considered as a risk factor for early-onset sepsis, while maternal age is not considered as a determining factors linked to the occurrence of NEOS in neonates [28, 29].

It the current study, we assessed the association between NEOS occurrence and some neonatal demographic factors. Based on the findings presented herein, a significant correlation was observed between neonatal sepsis and low Apgar score. In comparison, other studies suggested different first minute Apgar scores (< 7/5, 4 or 6) as an independent risk factor which was capable of increasing the risk of NEOS [30–32]. Moreover, our findings revealed that neonatal sepsis was significantly more common in males than females. As s previous study indicated, the male/female sex ratio has been found to be 1.3:1 among 344 neonates suffering from sepsis [33]. On the other hand, another study from Iran by Afsharpaiman et al. have demonstrated that although EOS was more common among males (24;54.5%) than females (20;45.5%), where there have been no significant differences between the two gender [34].

Overall, ample evidence has indicated that Vitamin D is capable of showing an impressive physiologic enhancement in maternal circulation. High prevalence rates of vitamin. D deficiency has been depicted previously in pregnant women, where can lead to negative outcomes for the mother and subsequently can affect infants’ short-term and long-term health. There is strong evidence that a number of mechanisms are potentially involved in observed correlations including immunomodulatory anti-inflammatory and metabolic effects of vitamin D; additionally, it can potentially play a pivotal role in epigenetic modifications of vitamin D - related genes. Whilst there is often controversial evidence that vitamin D has not been introduced as therapeutic modalities to selectively prevent pregnancy complications. However, it seems that maternal and neonatal supplies of vitamin D can provide a confirmatory approach to elucidate the events that contribute to the prevention of birth complications [11].

Our study should be considered in the light of some limitation, as the study could not evaluate our case group regarding blood culture and type of pathogen responsible sepsis and also the sample size was small. Another limitation of the study was the lack of evaluation of the possible relationship between maternal serum vitamin D concentrations with late-onset sepsis, where further studies are suggested in the near future. Therefore, further interventional randomized controlled trials and observational investigations could provide informative and beneficial data in order to prevent birth complications.

Moreover, further studies with larger sample size are strongly encouraged. Further development will need the establishment of evidence-based guidelines and novel markers for vitamin D supplementation [11].

## Conclusion

Our data suggested that the level of maternal serum Vitamin D was inversely correlated with neonatal sepsis occurrence and intake of vitamin D supplement during pregnancy could decrease the risk of early neonatal sepsis.

## Data Availability

The datasets used and analyzed during the current study are available from the corresponding author on reasonable request.

## Abbreviations

25 OHD: 25-hydroxyvitamin D3
CRP: C-reactive protein
DIC: Disseminated intravascular coagulation
IDDM: Insulin dependent diabetes mellitus
IL-6: Interleukin-6
NEOS: Neonatal early-onset sepsis
NICU: Neonatal Intensive Care Unit
